# Real-World Treatment Patterns, Clinical Outcomes, and Health Care Resource Utilization in Extensive-Stage Small Cell Lung Cancer in Canada

**DOI:** 10.3390/curroncol28040270

**Published:** 2021-08-13

**Authors:** Dylan E. O’Sullivan, Winson Y. Cheung, Iqra A. Syed, Daniel Moldaver, Mary Kate Shanahan, D. Gwyn Bebb, Christina Sit, Darren R. Brenner, Devon J. Boyne

**Affiliations:** 1 Department of Oncology, University of Calgary, Calgary, AB T2N 4N2, Canada; Winson.Cheung@albertahealthservices.ca (W.Y.C.); Gwyn.Bebb@albertahealthservices.ca (D.G.B.); darren.brenner@ucalgary.ca (D.R.B.); devon.boyne1@ucalgary.ca (D.J.B.); 2 Department of Community Health Sciences, University of Calgary, Calgary, AB T2N 4N2, Canada; 3 Oncology Outcomes Initiative, University of Calgary, Calgary, AB T2N 4N2, Canada; 4 AstraZeneca Canada, Mississauga, ON L4Y 1M4, Canada; iqra.syed@astrazeneca.com (I.A.S.); daniel.moldaver@astrazeneca.com (D.M.); marykate.shanahan@astrazeneca.com (M.K.S.); 5 Lung Cancer Canada, Toronto, ON M5H 2L3, Canada; ChristinaSit@lungcancercanada.ca

**Keywords:** population-based study, lines of therapy, chemotherapy, overall survival, real-world evidence

## Abstract

The prognosis for extensive-stage small cell lung cancer (ES-SCLC) is poor. Real-world evidence can highlight the unmet clinical need within this population. We conducted a population-based cohort study of ES-SCLC patients diagnosed in a large Canadian province (2010–2018) using electronic medical records and administrative claims data. In all, 1941 ES-SCLC patients were included, of which 476 (25%) were recurrent cases. Median age at diagnosis was 70 years (range: 39–94) and 50.2% were men. Of the 1941 ES-SCLC patients, 29.5% received chemotherapy and radiotherapy, 17.0% chemotherapy alone, 8.7% radiotherapy alone, and 44.8% received best supportive care. Chemotherapy was initiated by 46.5%, 8.5%, and 1.4% of first-, second-, and third-line patients, with lower uptake for recurrent cases. Median survival from first-, second-, and third-line chemotherapy was 7.82 months (95% CI: 7.50–8.22), 5.72 months (95% CI: 4.90–6.87), and 3.83 months (95% CI: 2.99–4.60). Among patients who received first-line therapy, the 2-year and 5-year survival was 7.3% (95% CI: 5.7–9.2) and 2.9% (95% CI: 1.8–4.5). In conclusion, initiation of first-line treatment in ES-SCLC was low with significant attrition in subsequent lines. These results underscore the need for effective front-line treatments and highlight the potential for novel therapies to improve patient outcomes.

## 1. Introduction

Lung cancer is the second most common type of cancer among both men and women in Canada, and small cell lung cancer (SCLC) accounts for approximately 12% of lung cancer cases [1]. SCLC is considered to be a very aggressive cancer that is often diagnosed late and has a high rate of recurrence and low survival [2]. SCLC is classified as either limited-stage (LS), where the cancer is contained to one side of the chest, or extensive-stage (ES), where the cancer has spread to tissue outside of the originally affected lung [2]. Approximately two-thirds of SCLC patients present with ES disease, while several patients with LS-SCLC experience recurrence [3]. SCLC can also be staged using the American Joint Committee on Cancer Tumor Node Metastasis (AJCC-TNM) system, which encompasses four broad stage groupings (I–IV) and various subgroupings.

First-line treatment for SCLC is often a platinum-based chemotherapy (cisplatin or carboplatin) in combination with etoposide, and radiation is considered for some patients [2]. Surgery is rarely a treatment option since the cancer has often spread at the time of diagnosis [2]. If recurrence occurs, topotecan or a combination of cyclophosphamide, doxorubicin, and vincristine (CAV) is typically administered [4]. The median survival for patients not receiving systemic therapy has been documented to be between 2 to 4 months [2], while patients receiving chemotherapy rarely exceed one year, with median survival across real-world studies from around the world ranging from 7.3 to 16 months [5]. In Canada, it is estimated that the 5-year survival for ES-SCLC patients who receive first-line chemotherapy is 3.7% [6]. There is an unmet need for effective and tolerable treatment options that extend survival for patients with ES-SCLC.

To address this unmet need of ES-SCLC patients, a better understanding of real-world treatment patterns and outcomes is required. To date, few studies have comprehensively examined multiple lines of therapy and associated outcomes in a large population-based real-world setting [5]. Of the studies that have examined multiple lines of therapy, the majority were contained to a single centre [7,8] and/or did not include untreated patients [8,9,10] or recurrent cases of ES-SCLC [7,8,9,10]. As such, there is a paucity of information on treatment patterns and long-term survival among patients with ES-SCLC in Canada. One study described the treatment patterns and survival of 276 ES-SCLC cases treated at a single tertiary cancer centre, but follow-up was limited and therefore long-term outcomes were not described [7]. The two other studies were a study in Manitoba that examined survival of ES-SCLC patients who received first-line chemotherapy [6] and a study conducted in Ontario that examined age-specific treatment patterns but combined LS and ES cases [11]. Since the majority of previous studies on this topic were not truly population based, the results may be subject to selection biases due to the exclusion of patients who were not referred to or treated at a tertiary/academic cancer centre. In addition, the inclusion of untreated patients and recurrent cases of ES-SCLC is important to address the full spectrum of disease trajectories. Finally, no previous studies in Canada have examined health care resource utilization among ES-SCLC patients.

To address this knowledge gap, the primary objective of this study was to comprehensively describe the treatment patterns and overall survival of patients with ES-SCLC in Alberta, Canada, using population-based data. A description of the demographic and clinical characteristics, an assessment of time to subsequent therapy, and an estimation of the health care resource utilization associated with ES-SCLC were secondary objectives.

## 2. Materials and Methods

This study was a retrospective longitudinal cohort study that leveraged the real-world, unselected population-level data in Alberta, Canada. The database included Alberta’s integrated provincial health care system, cancer registry, electronic health records, and laboratory and pathology results. Additional covariates were captured through the hospitalization discharge abstract database, physician billing claims, and the national ambulatory care reporting system databases maintained by the Alberta Government. The database covers 17 cancer centres (2 tertiary centres, 4 regional centres, and 11 community centres), which provides coverage of the entire population of Alberta (approximately 4.5 million residents).

The study population included all patients aged 18+ with a de novo diagnosis of ES-SCLC or a diagnosis of ES-SCLC after progression from LS-SCLC (recurrent) between January 2010 to December 2018. The cancer registry used to identify cases captures information on TNM stage rather than LS/ES. Analyses included all individuals with TNM stage IV disease, which was used as a proxy for ES disease. Patients who presented with early stage disease were classified as being recurrent if they received two plus cycles of chemotherapy more than one year after the date of the primary treatment, or initiated radiation therapy more than one year after the date of primary treatment, or died due to lung cancer [12]. The study population included all ES-SCLC patients whether referred or not referred to see an oncologist. Patients were followed until death or until December 2019, whichever came first.

The study measures included were patient demographics and clinical characteristics, treatment sequence patterns, and clinical outcomes such as overall survival. Baseline characteristics were only estimated for de novo ES-SCLC since the exact date of recurrence and information at the time of recurrence was not captured within the administrative datasets for recurrent cases. All baseline demographics and clinical characteristics were stratified by receipt of chemotherapy and where relevant by de novo/recurrent status. Continuous study measures were reported descriptively with mean and standard deviation (SD). Frequencies and percentages were used to document categorical measures of interest. All cell counts with fewer than 10 patients were suppressed (reported as <10 in tables) due to data privacy regulations. To compare the distribution of the baseline characteristics between those who initiated chemotherapy and those who did not, *p*-values corresponding to *t*-tests for continuous variables and chi-square tests for categorical variables are presented as are standardized mean differences (SMD) in which values >0.1 are indicative of an imbalance [13,14].

A Sankey diagram was generated to depict the relative sample sizes and proportions of patients receiving different therapies along the treatment trajectory from 1 L to 2 L. Since several treatment regimens were rare (<10 patients), these regimens were grouped together and classified as “other”. A list of treatments included in this category for each line of therapy is presented in Table S1. With respect to treatment duration, time on therapy was estimated as the time from initiation to the last chemotherapy cycle plus 21 days (typical duration of a cycle of chemotherapy) or until the initiation of the subsequent line of chemotherapy, whichever came first (patients were censored at death or end of study). Median time on therapy was estimated with the Kaplan–Meier method. Survival curves and median time-to-event were estimated via the Kaplan–Meier method for overall survival, 2-year survival, and 5-year survival. Analyses were conducted for all patients and also stratified by de novo and recurrent patients. Results were presented separately for de novo and recurrent patients when they differed considerably.

Health care resource utilization was quantified for hospitalizations (number of times and number of days), ambulatory care services (number of encounters overall, number of emergency encounters, and number of non-emergency encounters), cancer physician visits (number of visits overall and broken down by medical oncologist, radiation oncologist, general/family practitioner, or other cancer physicians), non-cancer practitioner visits (number of encounters and number of claims), and number of days of radiation therapy. The total number of events and the mean number of events per patient were estimated within each year of follow-up. Non-cancer practitioner encounters were estimated by the number of practitioner claims, in which multiple claims on the same day were treated as a single encounter.

## 3. Results

### 3.1. Patient Characteristics

A total of 1941 ES-SCLC patients were included in this study, with 1465 (75%) patients diagnosed with de novo ES-SCLC and 476 (25%) patients who initially presented with LS-SCLC but had evidence of a recurrence to ES-SCLC. Baseline patient demographics and clinical characteristics for de novo patients overall and stratified by receipt of chemotherapy (either alone or in combination with radiotherapy) or not (radiotherapy alone or only best supportive care) are presented in Table 1.

The average age of de novo patients in this study was 69.07 years of age (SD = 9.65), and there was an equal distribution of men and women. Relative to patients who initiated chemotherapy, patients who did not initiate chemotherapy were significantly older (*p* < 0.001; SMD = 0.469); had more comorbidities (*p* < 0.001; SMD = 0.306), including chronic pulmonary disease (p = 0.004; SMD = 0.153), cardiovascular disease (*p* < 0.001; SMD = 0.209), and renal disease (*p* < 0.003; SMD = 0.160); had worse performance status based on proxy measures (all *p* < 0.05; all SMD > 0.15); and were less likely to have metastases to the lymph nodes (*p* = 0.034; SMD = 0.116) but more likely to have metastases to the pleura (*p* = 0.001; SMD = 0.186) or bone marrow (*p* = 0.048; SMD = 0.111). Baseline patient demographics and clinical characteristics for de novo patients stratified by chemotherapy + radiation, chemotherapy alone, radiation alone, and best supportive care are presented in Table S2. Compared to patients who received both chemotherapy and radiation, patients who received chemotherapy alone or radiation alone were significantly older, had more comorbidities, and had worse performance status based on proxy measures.

### 3.2. Treatment Patterns

Of the 1941 ES-SCLC patients (de novo and recurrent), 573 (29.5%) received chemotherapy and radiotherapy, 330 (17.0%) chemotherapy alone, 168 (8.7%) radiotherapy alone, and 870 (44.8%) received only best supportive care (no active anti-cancer therapy). The majority of patients received chemotherapy and radiotherapy at an academic facility (85.3% and 88.0%, respectively). In total, 903 patients (46.5%) initiated first-line, 169 (8.7%) initiated second-line, and 28 (1.4%) initiated third-line chemotherapy (Table 2).

A total of 656 de novo ES-SCLC patients received radiotherapy, of which 350 (53.4%) had information available on the site of radiation. Among these 350 patients, 235 (67.1%) received thoracic radiation and 160 (45.7%) received brain radiation at some point during their treatment trajectory. The majority of individuals who were treated with thoracic radiation (176/235; 74.9%) or brain radiation (128/160; 80.0%) also received systemic therapy.

Among the 1199 de novo ES-SCLC cases who did not have a brain metastasis at diagnosis, 506 (42.2%) received radiation at some point during their treatment trajectory, whereby 115 (9.5%) received brain radiation, 157 (13.1%) received radiation to another site(s), and 234 (19.5%) were treated with radiation, but the specific site was unknown.

Among those who initiated first-line therapy, the majority of patients received either carboplatin plus etoposide (49.5%) or cisplatin plus etoposide (43.0%). The average time from diagnosis to first-line chemotherapy was 4.4 (SD = 5.7) weeks for carboplatin plus etoposide and 3.9 (SD = 3.5) weeks for cisplatin plus etoposide. The median time on first-line therapy was 15.0 weeks for carboplatin plus etoposide, 13.1 weeks for cisplatin plus etoposide, and 9.7 weeks for etoposide alone, respectively.

Of the 169 (8.7%) patients who initiated second-line therapy, the most common second-line therapies were carboplatin plus etoposide (52.7%) and CAV (24.9%). The median time on second-line therapy was 13.3 weeks for carboplatin plus etoposide, 7.4 weeks for CAV, and 9.0 weeks for topotecan, respectively.

**Figure 1:**
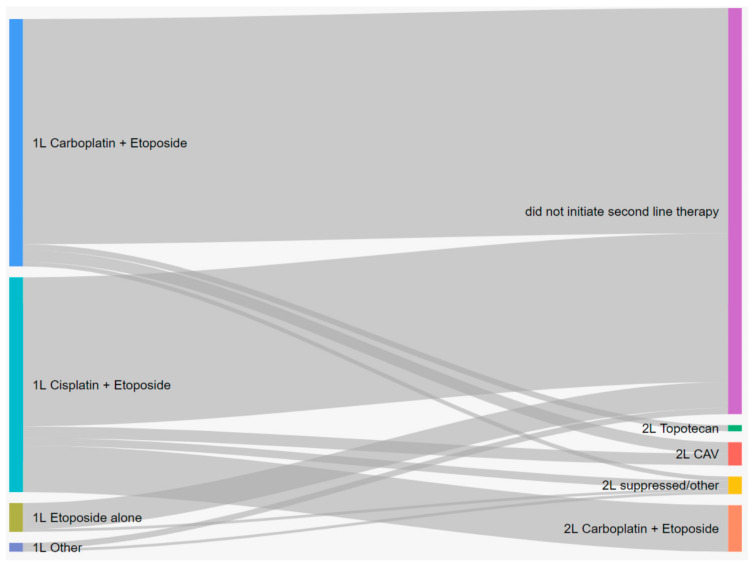
Treatment patterns of ES-SCLC patient in Alberta, Canada, from first-line to second-line therapy.

Of the 28 (1.4%) of patients who initiated third-line therapy, the majority of patients received CAV (38.5%) or topotecan (38.5%). Patients who received carboplatin plus etoposide for first-line therapy primarily received CAV for second-line therapy, while patients who received cisplatin plus etoposide for first-line therapy primarily received carboplatin plus etoposide for second-line therapy (Figure 1).

Treatment characteristics were significantly different between de novo and recurrent ES-SCLC cases (SMD = 0.809) (Table S3). A greater proportion of de novo cases (63.9%) initiated treatment in the form of radiation and/or chemotherapy relative to that in recurrent cases (28.4%). Among de novo cases, 54.6% received first-line chemotherapy, while only 21.0% of recurrent cases received first-line chemotherapy. In the first-line setting, recurrent cases primarily received carboplatin plus etoposide (Table S3).

### 3.3. Clinical Outcomes

**Figure 2:**
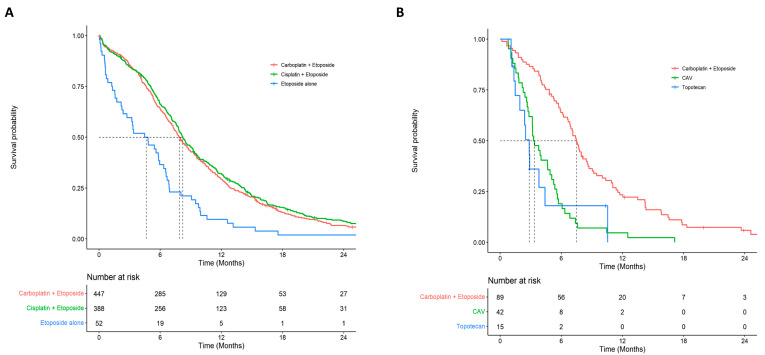
Overall survival by treatment for first- and second-line chemotherapy: (**A**) first-line overall survival by type of chemotherapy regimen; (**B**) second-line overall survival by type of chemotherapy regimen.

Median survival from diagnosis was considerably higher for patients who were treated with chemotherapy and radiotherapy (10.59 months; 95% CI: 10.03–11.61) compared to patients who received chemotherapy alone (5.65 months; 95% CI: 5.06–6.12), radiotherapy alone (3.02 months; 95% CI: 2.47–4.01), or those who received best supportive care (0.82 months; 95% CI: 0.72–0.92; log-rank *p*-value < 0.001). Median survival from first-line systemic therapy, second-line therapy, and third-line therapy were 7.82 months (95% CI: 7.50–8.22), 5.72 months (95% CI: 4.90–6.87), and 3.83 months (95% CI: 2.99–4.60), respectively. Among patients who received first-line therapy, the 2-year and 5-year survival was 7.3% (95% CI: 5.7–9.2) and 2.9% (95% CI: 1.8–4.5). The 2-year and 5-year survival for patients who received second-line therapy was 3.9% (95% CI: 1.7–9.0) and 1.3% (95% CI: 0.2–7.9). Survival curves for each type of chemotherapy within first- and second-line (up to 24 months) settings are presented in Figure 2. Extended survival curves (up to 120 months) are presented in Figure S1.

In the first-line setting, the median survival was higher for patients who received carboplatin plus etoposide (7.89 months; 95% CI: 7.33–8.65) or cisplatin plus etoposide (8.22 months; 95% CI: 7.76–8.65), and lower for patients who only received etoposide (4.64 months; 95% CI: 2.37–6.54). The median survival was higher for patients who received carboplatin plus etoposide (7.50 months; 95% CI: 6.67–8.61) and lower for patients who received CAV (3.34 months; 95% CI: 2.83–5.06) or topotecan (2.86 months; 95% CI: 1.94–NA) in the second-line setting. Median survival for patients who re-challenged with platinum-based chemotherapy was 7.69 months (95% CI: 6.87–9.04) and the median time between the start of first-line and second-line treatment for those patients was 11.9 weeks. Median survival did not vary considerably between de novo and recurrent cases by line of therapy or when stratified by treatment (Table S4). Among the patients who were diagnosed with ES-SCLC in 2010 (start of the cohort), 1.6% were alive at the end of follow-up, and all of these patients received a combination of chemotherapy and radiotherapy.

### 3.4. Health Care Resource Utilization

Health care resource utilization from initiation of first-line therapy by type of treatment (mean per patient) is presented in Table 3. Health care resource utilization among de novo patients who did not initiate chemotherapy and from the initiation of second-line therapy are presented in Tables S5 and S6.

## 4. Discussion

In relation to treatment rates, data showed that a considerable proportion of ES-SCLC patients only received best supportive care (36.1% for de novo cases and 71.6% for recurrent cases) and that survival from the time of diagnosis was less than a month for the de novo cases. A population-based study in the Netherlands observed a similarly high proportion of de novo patients who received only best supportive care (28%) [9] and their survival was also less than a month. In a single-centre study conducted in Calgary that consisted of a selected group of patients referred to a medical oncologist, the survival among patients who received best supportive of care (n = 38) was slightly higher at 1.7 months [7]. In this study, we found that patients who did not initiate chemotherapy were more likely to be older, have more comorbidities, and worse performance status. Future studies should examine patient characteristics of this patient population, and targeted screening approaches should be considered.

Of the patients who initiated chemotherapy (46.5%) in this study, only 18.7% initiated second-line therapy, and 3.0% initiated third-line therapy. The low initiation of second- and third-line therapy is consistent with other population-based studies that have comprehensively examined treatment patterns in ES-SCLC conducted in the Netherlands, Sweden, Germany, and Canada [7,8,9,10]. The low initiation of subsequent lines of therapy highlights the importance of novel first-line therapies that are well tolerated and have a durable response. Immunotherapies (atezolizumab [15] and durvalumab [16]) in addition to platinum–etoposide combination therapy have been evaluated in clinical trials and have shown significant improvements in survival outcomes [17]. Of the patients who initiated first-line therapy in this study, 92.5% received a platinum–etoposide combination therapy, and this is consistent with several studies conducted in Europe and Canada [7,8,9,10]. It is reasonable to suppose that the majority of ES-SCLC patients well enough to embark on platinum-doublet chemotherapy would be eligible to receive these novel first-line therapies in a real-world setting.

In the first-line setting, the majority of patients received either carboplatin plus etoposide (49.5%) or cisplatin plus etoposide (43.0%), which is in line with Canadian guidelines that recommend platinum-based doublet chemotherapy. The similar preference between cisplatin and carboplatin is unique, since elsewhere in the world there seems to be a considerably larger preference for carboplatin over cisplatin [8,9,10]. However, the use of carboplatin was higher in this study compared to that in a study conducted on an earlier cohort in Manitoba [6], which could be evidence that the balance between cisplatin and carboplatin may slowly be shifting towards carboplatin in Canada. In contrast, this could also be due to the inclusion of recurrent cases in this study, which were more likely to receive carboplatin plus etoposide. Among patients who received first-line chemotherapy, we observed a median overall survival of 7.8 months with similar survival between platinum–etoposide combination therapies (carboplatin plus etoposide vs. cisplatin plus etoposide). Overall survival was consistent with studies conducted in the Netherlands and Sweden, which found median overall survival of 7.4 and 7.1 months for patients who initiated first-line chemotherapy [8,9]. In a prospective cohort study conducted in Germany, the overall survival was higher at 10.7 months [10]. However, the German study only included patients who received at least one palliative line of treatment, which would likely exclude patients with early discontinuation who would have shorter survival durations. A single-centre study in Calgary, Alberta, observed a slightly higher survival of approximately 9 months [7]. In this study, we also observed that patients who received both chemotherapy and radiation had better overall survival than patients who only received chemotherapy. This is likely due in part to the additional treatment (radiation) but also due to less aggressive disease and likely better performance status that makes these patients eligible for the additional treatment.

In the second-line setting, patients who received carboplatin plus etoposide for first-line therapy primarily received CAV, while patients who received cisplatin plus etoposide for first-line therapy primarily received carboplatin plus etoposide. Over half of the patients who initiated second-line treatment re-challenged with a platinum-based chemotherapy, which is consistent with studies conducted in the Netherlands [9] and Sweden [8], while patients in the German study were typically administered topotecan as a second-line therapy [10]. In this study, survival was considerably higher for patients who received carboplatin plus etoposide compared to that for those who received both CAV and topotecan in the second-line setting, which is consistent with previous reports [8,18]. Overall, the survival outcomes for patients who initiated second- and third-line therapy was 5.7 and 3.8 months, which illustrates the difficulty in treating and studying patients in second. and third-line settings. With advent of novel first-line therapies and the low uptake of third-line therapies, there is a clear demand for continued research into novel second-line therapies.

A strength of this investigation is that the long follow-up period allowed for an examination of long-term survival outcomes. Among patients who received first-line chemotherapy, the 5-year survival was 2.9%, which was slightly lower but in the range of a smaller population-based study conducted in Manitoba (3.7%) [6]. In the second-line setting, the 5-year survival was lower at 1.3%. Interestingly, of the patients who entered the cohort in the first year of the study, 1.6% remained alive at the end of follow-up (>9 years), and all of the patients received a combination of chemotherapy and radiotherapy. These results indicate that while 5-year survival remains low, some patients had a long-term response to therapy. The introduction of novel therapies into the real world has the potential to increase the percentage of patients who have a durable response to therapy. Studies that examine characteristics of patients who respond well to therapy, including molecular markers, could yield important insights for future treatments and clinical management of ES-SCLC.

To our knowledge, this is the largest and most up-to-date population-based study to comprehensively examine treatment patterns, clinical outcomes, and health care resource utilization in extensive-stage small cell lung cancer in Canada. Strengths of this investigation include the quality of chemotherapy data, which are routinely captured in electronic medical records; the identification of cancer cases through linkage with the Alberta Cancer Registry and not just captured through claim algorithms; little missing data; and short lag period between the current calendar date and the end of follow-up. In addition, both untreated patients and patients with recurrent cases of ES-SCLC were included, which is rarely done in other real-world studies. Finally, due to the comprehensive population-based nature of the data (complete population inclusion), the results from this study are generalizable to Alberta and other provinces of Canada.

The limitations of this investigation should also be highlighted. This study relied upon administrative data, which do not routinely capture some important clinical covariates that may be of interest such as performance status, smoking history, disease progression, and low-grade treatment toxicity. In addition, we were unable to robustly examine survival by site of radiation due to missing data. The Alberta Cancer Registry does not capture recurrent cases of cancer, which necessitated the use of an administrative data algorithm. This may have led to the misclassification of some of the recurrent cases included in this study. Additionally, individuals were classified as having ES disease based on having TNM stage IV disease, which may have led to some misclassification. However, we expect such misclassification to be minimal, given that an estimated 95% of ES-SCLC cases are TNM stage IV [19]. Baseline characteristics were only estimated for de novo ES-SCLC since the exact date of recurrence and information at the time of recurrence was not captured within the administrative datasets for recurrent cases. We were unable to estimate time-corrected rates of health care resource utilization since the rates were not constant over the duration of follow-up (e.g., some patients may live 1 month and see a physician almost every day, and this rate would be extrapolated over an entire year, leading to artificially high rates). Lastly, statements regarding the comparative efficacy or safety of therapies cannot be made on the basis of these results since this study was descriptive in nature and did not attempt to control for confounding, immortal time, and other sources of bias.

## 5. Conclusions

In this population based study of ES-SCLC patient in Canada, we observed that the initiation of first-line treatment was low with significant attrition in subsequent lines. It was evident that patients who received chemotherapy had greater survival compared to those who did not. However, survival was modest for all lines of therapy. These results underscore the need for earlier detection and effective front-line therapeutic options, and highlight the potential for novel therapies to improve patient outcomes in the real-world. 

## Figures and Tables

**Table 1:** Baseline demographics and clinical characteristics of the de novo extensive stage small cell lung cancer patients.

Variable	Overall	Chemotherapy **	No Chemotherapy ***	*p*-Value	SMD
	(*n* = 1465)	(*n* = 803)	(*n* = 662)		
**Demographics**					
Age, years (mean (SD))	69.07 (9.65)	67.08 (9.15)	71.50 (9.70)	<0.001	0.469
<60 years (%)	274 (18.8)	181 (22.5)	93 (14.0)	<0.001	0.221
≥60 years (%)	1191 (81.3)	622 (77.5)	569 (86.0)		
Male (%)	741 (50.6)	414 (51.6)	327 (49.4)	0.441	0.043
**Socioeconomic Status**					
Urban residence (%)	1138 (77.7)	634 (79.0)	504 (76.1)	0.22	0.068
Neighbourhood annual household income in Canadian dollars (mean (SD))	36,073.50 (13,518.67)	36,571.78 (13,359.62)	35,469.09 (13,694.91)	0.12	0.082
Categories of neighbourhood annual household income in Canadian dollars (%)				0.051	0.146
0–25,000	131 (8.9)	62 (7.7)	69 (10.4)		
25,000–35,000	703 (48.0)	372 (46.3)	331 (50.0)		
35,000–45,000	413 (28.2)	238 (29.6)	175 (26.4)		
>45,000	218 (14.9)	131 (16.3)	87 (13.1)		
Proportion of neighbourhood residents who achieved a high school education or greater (mean (SD))	0.74 (0.11)	0.74 (0.11)	0.73 (0.11)	0.09	0.089
Categories of neighbourhood education (%)				0.333	0.097
0.00–0.60	166 (11.3)	88 (11.0)	78 (11.8)		
0.60–0.70	328 (22.4)	174 (21.7)	154 (23.3)		
0.70–0.80	510 (34.8)	272 (33.9)	238 (36.0)		
>0.80	461 (31.5)	269 (33.5)	192 (29.0)		
**Comorbidity**					
Charlson comorbidity index (%)				<0.001	0.306
0	601 (41.0)	369 (46.0)	232 (35.0)		
1	432 (29.5)	238 (29.6)	194 (29.3)		
2	225 (15.4)	110 (13.7)	115 (17.4)		
3	105 (7.2)	52 (6.5)	53 (8.0)		
≥4	102 (7.0)	34 (4.2)	68 (10.3)		
Chronic obstructive pulmonary disease (%)	532 (36.3)	265 (33.0)	267 (40.3)	0.004	0.153
Diabetes (%)	325 (22.2)	169 (21.0)	156 (23.6)	0.275	0.061
Cardiovascular disease (%)	271 (18.5)	119 (14.8)	152 (23.0)	<0.001	0.209
Renal disease (%)	66 (4.5)	24 (3.0)	42 (6.3)	0.003	0.16
Liver disease (%)	56 (3.8)	26 (3.2)	30 (4.5)	0.251	0.067
Connective tissue disease (%)	27 (1.8)	16 (2.0)	11 (1.7)	0.784	0.025
**Indicators of health**					
Prior cancer	123 (8.4)	59 (7.3)	64 (9.7)	0.134	0.083
No. of hospitalizations within 1 year prior to diagnosis * (%)				<0.001	0.326
0	1222 (83.4)	712 (88.7)	510 (77.0)		
1	153 (10.4)	64 (8.0)	89 (13.4)		
2	53 (3.6)	15 (1.9)	38 (5.7)		
≥3	37 (2.5)	12 (1.5)	25 (3.8)		
No. of ambulatory care encounters within the year prior to diagnosis * (mean (SD))	4.27 (9.90)	3.58 (7.88)	5.10 (11.84)	0.004	0.15
No. of health practitioner encounters within the year prior to diagnosis * (mean (SD))	13.46 (14.73)	11.89 (10.93)	15.35 (18.14)	<0.001	0.231
**Metastatic Sites**					
Number of metastatic sites at diagnosis				0.269	0.133
1	548 (37.4)	285 (35.5)	263 (39.7)		
2	452 (30.9)	265 (33.0)	187 (28.2)		
3	251 (17.1)	141 (17.6)	110 (16.6)		
4	126 (8.6)	70 (8.7)	56 (8.5)		
≥5	86 (5.9)	41 (5.1)	45 (6.8)		
Missing	2 (0.1)	1 (0.1)	1 (0.2)		
Sites of metastasis at diagnosis					
Hepatic	695 (47.4)	363 (45.2)	332 (50.2)	0.067	0.099
Pleura	674 (46.0)	336 (41.8)	338 (51.1)	0.001	0.186
Osseous	445 (30.4)	260 (32.4)	185 (27.9)	0.075	0.097
Lymph nodes	282 (19.2)	171 (21.3)	111 (16.8)	0.034	0.116
Brain	266 (18.2)	148 (18.4)	118 (17.8)	0.817	0.016
Adrenals	262 (17.9)	151 (18.8)	111 (16.8)	0.345	0.053
Pulmonary	199 (13.6)	119 (14.8)	80 (12.1)	0.149	0.08
Peritoneum	57 (3.9)	38 (4.7)	19 (2.9)	0.089	0.097
Bone marrow	33 (2.3)	12 (1.5)	21 (3.2)	0.048	0.111

* Proxy measures for performance status. Abbreviations: SD = standard deviation; SMD = standardized mean difference. ** Includes patients who received chemotherapy alone or in combination with radiotherapy; *** includes patients who received radiotherapy alone or only standard best care.

**Table 2:** Time on treatment and survival outcomes of ES-SCLC patients by treatment type and line of therapy.

Variable	Estimate (%)	Time on Therapy	Median Survival	2-Year Survival	5-Year Survival
	*n* = 1941	Median (KM)	(95% CI)	(95% CI)	(95% CI)
**1L Chemotherapy**	903 (46.5)				
Carboplatin + Etoposide	447 (49.5)	15.0	7.89 (7.33–8.65)	0.066 (0.046–0.095)	0.022 (0.010–0.050)
Cisplatin + Etoposide	388 (43.0)	13.1	8.22 (7.76–9.14)	0.087 (0.063–0.121)	0.041 (0.024–0.071)
Etoposide alone	52 (5.8)	9.7	4.64 (2.37–6.54)	0.019 (0.003–0.134)	NA
Other/Suppressed	16 (1.8)	-	-	-	-
**2L Chemotherapy**	169 (8.7)				
Carboplatin + Etoposide	89 (52.7)	13.3	7.50 (6.67–8.61)	0.059 (0.025–0.143)	0.020 (0.003–0.123)
CAV	42 (24.9)	7.4	3.34 (2.83–5.06)	NA	NA
Topotecan	15 (8.9)	9.0	2.86 (1.94–NA)	NA	NA
Other/Suppressed	23 (13.6)	-	-	-	-
**3L Chemotherapy**	28 (1.4)				
CAV	10 (38.5)	7.1	2.89 (1.32–NA)	NA	NA
Topotecan	10 (38.5)	13.6	3.83 (2.24–NA)	NA	NA
Other/Suppressed	8 (23.0)	-	-	-	-

Abbreviations: CAV = combination of cyclophosphamide, doxorubicin, and vincristine; CI = confidence interval; KM = Kaplan–Meier; L = line. Time on therapy is measured in weeks; overall survival is measured in months; 2-year and 5-year survival is expressed as a probability.

**Table 3:** Average health care resource utilization per patient per year from first-line therapy among de novo and recurrent ES-SCLC cases by treatment type.

First-Line Therapies		Etoposide Alone	Other	Platinum + Etoposide				
Construct	Outcome	Year 1 (*n* = 52)	Year 1 (*n* = 16)	Year 1 (*n* = 835)	Year 2 (*n* = 253)	Year 3 (*n* = 58)	Year 4 (*n* = 27)	Year 5 (*n* = 17)
Hospitalizations	No. of Hospitalizations	1.06	1.50	1.49	0.98	0.60	0.44	0.88
	No. of Days Hospitalized	15.42	16.75	16.94	11.57	9.57	6.26	10.35
Ambulatory Care Services	No. of Encounters	5.75	6.69	7.54	4.49	4.74	3.78	4.59
	No. of Emergency Encounters	1.60	3.75	2.98	1.67	1.34	1.11	1.47
	No. of Non-Emergency Encounters	4.15	2.94	4.56	2.82	3.40	2.67	3.12
Cancer Physician Visits	No. of Visits	5.02	8.50	11.00	5.18	4.50	3.48	3.29
	No. of Medical Oncologist Visits	4.10	5.69	7.98	4.01	3.81	3.30	3.18
	No. of Radiation Oncologist Visits	0.65	1.38	2.02	0.77	0.66	<10	<10
	No. of General/Family Practitioner Visits	<10	0.81	0.54	0.17	<10	<10	<10
	No. of Other Cancer Physician Visits	0.19	0.63	0.47	0.23	<10	<10	<10
Non-Cancer Practitioner Visits	No. of Encounters	28.54	26.06	26.51	20.77	19.81	17.33	18.00
	No. of Claims	50.17	52.81	49.79	38.45	35.17	32.48	40.24
Radiation Therapy	No. of Days of Therapy	1.50	6.50	10.15	3.11	1.24	0.44	<10
Chemotherapy Cycles	No. of Cycles	3.52	8.69	9.73	2.47	1.52	0.59	<10

Note: Year 1 corresponds to 0–12 months; Year 2 corresponds to 12–24 months, etc. Abbreviation: No. = number.

## Data Availability

Aggregate-level data presented in this study are available on request from the corresponding author. Individual-level data are not publicly available due to Canadian data privacy laws governing personal health information.

## Supplementary Materials

The following are available online at https://www.mdpi.com/article/10.3390/curroncol28040270/s1, Figure S1: Long-term overall survival by treatment for first- and second-line chemotherapy. (A) Long-term first-line overall survival by type of chemotherapy regimen. (B) Long-term second-line overall survival by type of chemotherapy regimen, Table S1: List of rare treatment regimens (<10 patients) included in the “other” category for each line of therapy., Table S2: Baseline demographics and clinical characteristics of the de novo extensive stage small cell lung cancer patients stratified by chemotherapy + radiation, chemotherapy alone, radiation alone, and best supportive care., Table S3: Treatment characteristics stratified by de novo and recurrent ES-SCLC cases., Table S4: Overall survival of de novo and recurrent ES-SCLC cases by line of therapy and treatment, Table S5: Average health care resource utilization per patient per year among de novo ES-SCLC patients who did not initiate chemotherapy, Table S6: Average health care resource utilization per patient per year from second line therapy among de novo and recurrent ES-SCLC patients.
