# Supplementary material for: Real-World Treatment Patterns, Clinical Outcomes, and Health Care Resource Utilization in Extensive-Stage Small Cell Lung Cancer in Canada
Source: Curr Oncol. 2021 Aug 13;28(4):3091–103. doi: 10.3390/curroncol28040270 (PMC8395392; doi:10.3390/curroncol28040270)
Supplement: Supplementary file 1 [file curroncol-28-00270-s001.zip › curroncol-1329379-supplementary.pdf]

Supplementary Materials

Table S1. List of rare treatments regimens (< 10 patients) included in the "other" category for each line of therapy.

First Line	Second Line	Third Line
•CAV	•Cisplatin + Etoposide	•Carboplatin + Etoposide
•Topotecan alone	•Etoposide alone	•Cisplatin + Etoposide
•Carboplatin + Pemetrexed	•Carboplatin + Etoposide + Durvalumab + Tremelimumab	•Etoposide alone
•Carboplatin + Vinorelbine	•Cisplatin + Etoposide + Durvalumab	•Cisplatin + Etoposide + Durvalumab
•Carboplatin + Paclitaxel	•Carboplatin + Doxorubicin	•Gefitinib
•Capecitabine + Temozolomide	•Capecitabine + Temozolomide	•Ipilimumab + nivolumab
•Denosumab	•Pemetrexed	
•Gefitinib	•Denosumab	
	•Irinotecan	

Table S2. Baseline demographics and clinical characteristics of the de novo extensive stage small cell lung cancer patients stratified by chemotherapy + radiation, chemotherapy alone, radiation alone, and best supportive care.

Variable	Overall (n = 1465)	Chemo + Rad (n = 523)	Chemo Alone (n = 280)	Rad Alone (n = 133)	No Chemo or Rad (n = 529)	P-value	SMD
**Demographics**							
Age, years (mean [SD])	69.07 (9.65)	66.24 (9.12)	68.65 (9.01)	68.74 (9.90)	72.19 (9.53)	<0.001	0.319
≥ 60 years (%)	1191 (81.3)	391 (74.8)	231 (82.5)	107 (80.5)	462 (87.3)	<0.001	0.171
Male (%)	741 (50.6)	252 (48.2)	162 (57.9)	70 (52.6)	257 (48.6)	0.042	0.111
**Socioeconomic Status**							
Urban Residence (%)	1138 (77.7)	422 (80.7)	212 (75.7)	99 (74.4)	405 (76.6)	0.208	0.078
Neighbourhood Annual Household Income (mean[SD])	36073.50 (13518.67)	37057.66 (14672.93)	35664.22 (10438.85)	35728.49 (13807.24)	35403.87 (13678.89)	0.22	0.062
Categories Neighbourhood Annual Household Income (%)						0.327	0.104
0-<35k	834 (56.9)	279 (53.3)	155 (55.4)	80 (60.2)	320 (60.5)		
35-<45	413 (28.2)	157 (30.0)	81 (28.9)	37 (27.8)	138 (26.1)		
≥ 45k	218 (14.9)	87 (16.6)	44 (15.7)	16 (12.0)	71 (13.4)		
Proportion of Neighbourhood Residents who achieved a Highschool Education or Greater (mean[SD])	0.74 (0.11)	0.75 (0.11)	0.73 (0.11)	0.73 (0.11)	0.73 (0.11)	0.173	0.076
Categories of Neighbourhood Education (%)						0.502	0.114
0.00-<0.60	166 (11.3)	53 (10.1)	35 (12.5)	14 (10.5)	64 (12.1)		
0.60-<0.70	328 (22.4)	113 (21.6)	61 (21.8)	35 (26.3)	119 (22.5)		
0.70-<0.80	510 (34.8)	171 (32.7)	101 (36.1)	44 (33.1)	194 (36.7)		
0.80+	461 (31.5)	186 (35.6)	83 (29.6)	40 (30.1)	152 (28.7)		
**Comorbidity[1]**							
Charlson Comorbidity Index (%)						<0.001	0.277
0	601 (41.0)	268 (51.2)	101 (36.1)	50 (37.6)	182 (34.4)		
1	432 (29.5)	150 (28.7)	88 (31.4)	34 (25.6)	160 (30.2)		
2	225 (15.4)	64 (12.2)	46 (16.4)	30 (22.6)	85 (16.1)		

≥ 3	207 (14.1)	41 (7.8)	45 (16.1)	19 (14.3)	102 (19.3)		
Chronic Obstructive Pulmonary Disease (%)	532 (36.3)	155 (29.6)	110 (39.3)	53 (39.8)	214 (40.5)	0.001	0.116
Diabetes (%)	325 (22.2)	96 (18.4)	73 (26.1)	30 (22.6)	126 (23.8)	0.052	0.098
Cardiovascular Disease (%)	271 (18.5)	61 (11.7)	58 (20.7)	24 (18.0)	128 (24.2)	<0.001	0.177
**Indicators of health**[1,*]							
Hospitalized Within Year Prior to Diagnosis* (%)	243 (16.6)	47 (9.0)	44 (15.7)	25 (18.8)	127 (24.0)	<0.001	0.221
No. of Ambulatory Care Encounters Within Year Prior to Diagnosis *(mean [SD])	4.27 (9.90)	3.18 (6.23)	4.33 (10.25)	2.86 (3.96)	5.66 (13.04)	<0.001	0.172
No. of Health Practitioner Encounters Within Year Prior to Diagnosis *(mean [SD])	13.46 (14.73)	10.98 (9.79)	13.59 (12.64)	12.01 (12.54)	16.19 (19.21)	<0.001	0.201
**Metastatic Sites**							
Number of Metastatic Sites at Diagnosis						0.21	0.158
1	548 (37.4)	197 (37.7)	88 (31.4)	51 (38.3)	212 (40.1)		
2	452 (30.9)	172 (32.9)	93 (33.2)	39 (29.3)	148 (28.0)		
3	251 (17.1)	86 (16.4)	55 (19.6)	24 (18.0)	86 (16.3)		
≥ 4	212 (14.5)	68 (13.0)	43 (15.4)	18 (13.5)	83 (15.7)		
Missing	2 (0.1)	0 (0.0)	1 (0.4)	1 (0.8)	0 (0.0)		
Sites of Metastasis at Diagnosis[1]							
Hepatic	695 (47.4)	208 (39.8)	155 (55.4)	49 (36.8)	283 (53.5)	<0.001	0.235
Pleura	674 (46.0)	212 (40.5)	124 (44.3)	46 (34.6)	292 (55.2)	<0.001	0.223
Osseous	445 (30.4)	177 (33.8)	83 (29.6)	55 (41.4)	130 (24.6)	<0.001	0.196
Lymph nodes	282 (19.2)	103 (19.7)	68 (24.3)	19 (14.3)	92 (17.4)	0.048	0.138
Brain	266 (18.2)	106 (20.3)	42 (15.0)	44 (33.1)	74 (14.0)	<0.001	0.254
Adrenals	262 (17.9)	99 (18.9)	52 (18.6)	27 (20.3)	84 (15.9)	0.483	0.059
Pulmonary	199 (13.6)	77 (14.7)	42 (15.0)	14 (10.5)	66 (12.5)	0.446	0.078

[1] The number of individuals with concurrent renal, liver, and connective tissue disease as well as the number of individuals with prior cancer and number of individuals with peritoneum or bone marrow metastases were suppressed due to small cell counts

*Proxy measures for performance status

Abbreviations: SD = standard deviation; SMD = standardised mean difference.

Table S3. Treatment characteristics stratified by de novo and recurrent ES-SCLC cases.

Variable (%)	De novo (n=1465)	Recurrent (n=476)	P-Value	SMD
**Overall Treatment**			<0.001	0.809
Chemotherapy + radiotherapy	523 (35.7)	50 (10.5)		
Chemotherapy alone	280 (19.1)	50 (10.5)		
Radiotherapy alone	133 (9.1)	35 (7.4)		
No chemotherapy or radiotherapy	529 (36.1)	341 (71.6)		
**First-line Chemotherapy**			<0.001	0.52
Carboplatin + Etoposide	388 (48.3)	59 (59.0)		
Cisplatin + Etoposide	362 (45.1)	26 (26.0)		

Table S4. Overall survival of de novo and recurrent ES-SCLC cases by line of therapy and treatment.

Variable	Median Survival (95% CI)	2-Year Survival (95% CI)	5-Year Survival (95% CI)
**1L Chemotherapy**			
Overall			
De novo	7.76 (7.27-8.15)	0.071 (0.055-0.092)	0.029 (0.018-0.047)
Recurrent	8.52 (7.76-10.95)	0.087 (0.046-0.166)	NA
Carboplatin + Etoposide			
De novo	7.66 (7.23-8.28)	0.066 (0.045-0.098)	0.021 (0.009-0.050)
Recurrent	9.57 (7.79-11.74)	0.068 (0.026-0.175)	NA
Cisplatin + Etoposide			
De novo	8.22 (7.63-9.14)	0.085 (0.061-0.121)	0.041 (0.024-0.072)
Recurrent	8.38 (7.63-12.53)	0.103 (0.031-0.342)	NA
**2L Chemotherapy**			
Overall			
De novo	5.72 (4.83-7.07)	0.071 (0.055-0.092)	0.029 (0.018-0.047)
Recurrent	6.12 (4.34-12.53)	0.087 (0.046-0.166)	NA

Table S5. Average health care resource utilization per patient per year among de novo ES-SCLC patients who did not initiate chemotherapy.

Construct	Outcome	Year 1 (n=662)	Year 2 (n=22)
Hospitalizations	No. of Hospitalizations	1.14	0.59
	No. of Days Hospitalized	15.39	19.55
Ambulatory Care Services	No. of Encounters	3.09	5.32
	No. of Emergency Encounters	1.18	1.00
	No. of Non-Emergency Encounters	1.91	4.32
Cancer Physician Visits	No. of Visits	2.23	1.68
	No. of Medical Oncologist Visits	1.14	0.73
	No. of Radiation Oncologist Visits	0.51	<10
	No. of General/Family Practitioner Visits	0.15	<10
	No. of Other Cancer Physician Visits	0.42	0.50
Non-Cancer Practitioner Visits	No. of Encounters	20.74	26.41
	No. of Claims	43.60	49.73
Radiation Therapy	No. of Days of Therapy	1.76	0.91
Chemotherapy Cycles	No. of Cycles	-	-

Table S6. Average health care resource utilization per patient per year from second line therapy among de novo and recurrent ES-SCLC patients.

Construct	Outcome	Year 1 (n=169)	Year 2 (n=28)
Hospitalizations	No. of Hospitalizations	1.27	1.11
	No. of Days Hospitalized	13.01	14.00
Ambulatory Care Services	No. of Encounters	6.59	3.68
	No. of Emergency Encounters	2.63	1.57
	No. of Non-Emergency Encounters	3.96	2.11
Cancer Physician Visits	No. of Visits	8.72	3.68
	No. of Medical Oncologist Visits	6.08	2.89
	No. of Radiation Oncologist Visits	1.79	0.64
	No. of General/Family Practitioner Visits	0.44	<10
	No. of Other Cancer Physician Visits	0.41	<10
Non-Cancer Practitioner Visits	No. of Encounters	21.25	22.54
	No. of Claims	41.86	41.36
Radiation Therapy	No. of Days of Therapy	5.99	2.32
Chemotherapy Cycles	No. of Cycles	7.40	2.04

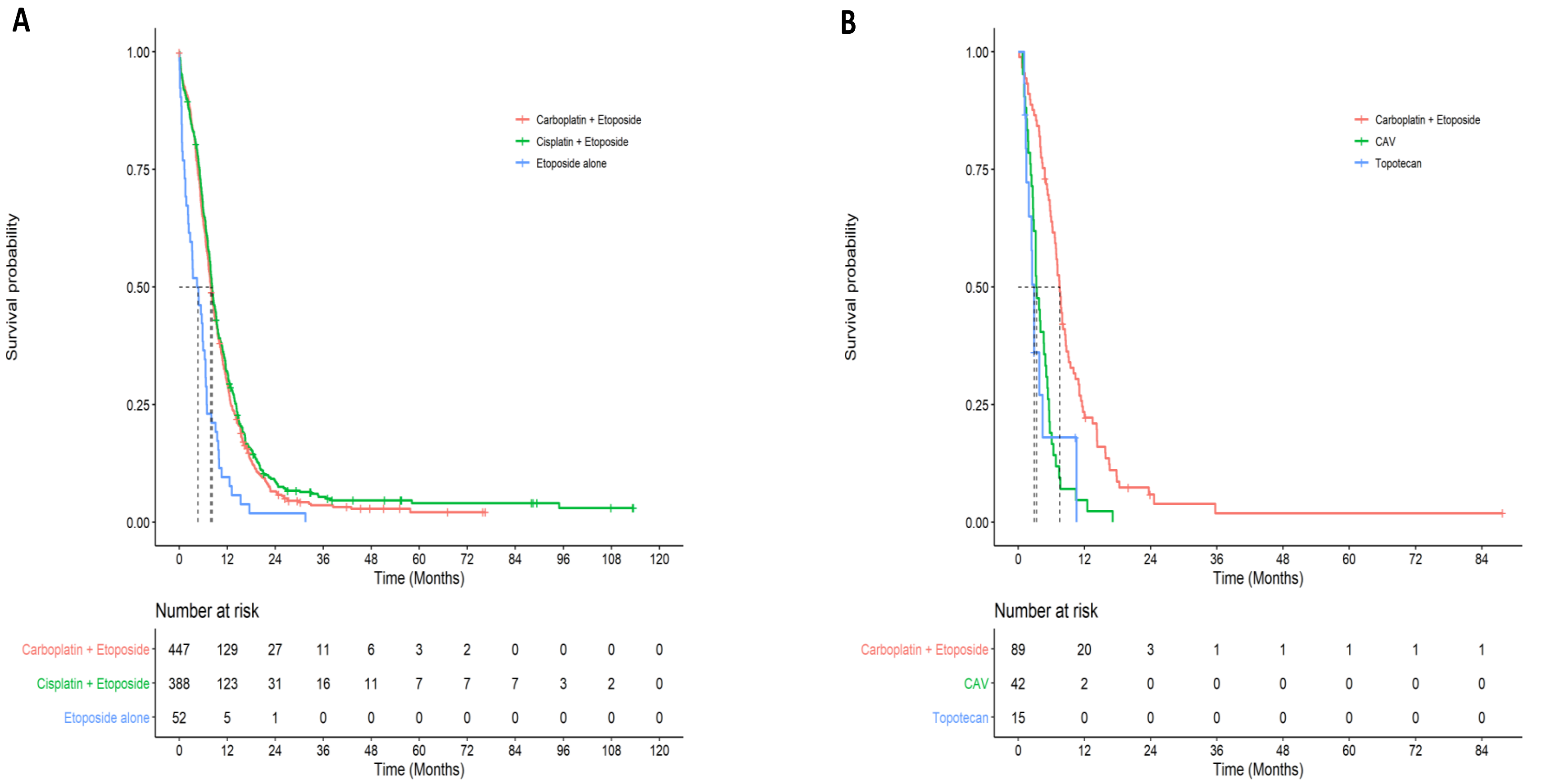

Figure S1. Long-term overall survival by treatment for first and second-line chemotherapy. **(A)** Long-term first-line overall survival by type of chemotherapy regimen. **(B)** Long-term second-line overall survival by type of chemotherapy regimen.
